# Part I: Dynamics of Recovery: A Meta-Synthesis Exploring the Nature of Mental Health and Substance Abuse Recovery

**DOI:** 10.3390/ijerph18157761

**Published:** 2021-07-22

**Authors:** Trude Klevan, Rose-Marie Bank, Marit Borg, Bengt Karlsson, Vibeke Krane, Esther Ogundipe, Randi Semb, Mona Sommer, Rolf Sundet, Knut Tore Sælør, Siw Heidi Tønnessen, Hesook Suzie Kim

**Affiliations:** Department of Health, Social and Welfare Studies, Faculty of Health and Social Sciences, University of South-Eastern Norway (USN), 3040 Drammen, Norway; rose-marie.bank@usn.no (R.-M.B.); marit.borg@usn.no (M.B.); bengt.karlsson@usn.no (B.K.); vibeke.krane@usn.no (V.K.); esther.ogundipe@usn.no (E.O.); randi.semb@usn.no (R.S.); mona.sommer@usn.no (M.S.); rolf.sundet@usn.no (R.S.); knut.tore.salor@usn.no (K.T.S.); siw.h.tonnessen@usn.no (S.H.T.); hsuziekim@comcast.net (H.S.K.)

**Keywords:** recovery, meta-synthesis, mental health and substance abuse, person–context, dynamics

## Abstract

Recovery-oriented care has become a leading vision across countries. To develop services and communities in more recovery-oriented directions, enhanced understandings of recovery in terms of personal and social contexts are important prerequisites. The aim of this study is to explore the nature and characteristics of the experiences of recovery. The method used is a form of qualitative meta-synthesis that integrates the findings from multiple qualitative studies published by one research group. Twenty-eight empirical papers with a focus on recovery as personal and contextual experiences were included in this meta-synthesis. Five meta-themes were developed: (a) being normal, (b) respecting and accepting oneself, (c) being in control, (d) recovery as intentional, and (e) recovery as material and social. The themes describe how recovery encompasses dynamics between personal experiences and contextual dimensions. This meta-synthesis consolidated an understanding of recovery as dynamics of the self and others, and as dynamics of the self and material resources. This understanding of recovery suggests the need to work not only with the person, but also with families, networks, social systems, and local communities, thus developing mental health and substance abuse services in more collaborative, open-ended, and context-sensitive directions.

## 1. Introduction

Within the transformation of mental health and addiction services towards more care provision in the community, recovery-oriented care has become a leading vision across countries [1,2]. This shift has led to a demand for more multifaceted knowledge of how living and dealing with mental health and/or substance abuse issues can be experienced and what truly helpful support may involve. However, the concept of recovery remains contested. Definitions and understandings vary across contexts and disciplines, spanning from those derived from the original roots of the recovery movement to versions developed within a clinical paradigm [1,3].

Tuffour distinguishes between clinically oriented and consumer-oriented definitions of recovery [3]. The former is described as considering recovery from a disease perspective, originating from the historical context of clinical research. From this perspective, recovery is understood as a clinical outcome, measured through aspects such as symptom decrease and improved functional status [4,5]. Clinical recovery is understood as best determined by professional experts, and commonly involves using standard assessment tools [6]. By contrast, rather than focusing on the absence of symptoms as the ultimate goal and the need for professionals to provide treatment, consumer-oriented understandings of recovery focus on regaining control over one’s life through one’s own efforts. Recovery is understood as non-linear, personal, social, and contextual processes through which people strive to overcome their difficulties over time, with or without support from private and/or professional networks [7,8,9,10]. Inclusion in community life with the ability to participate in meaningful activities and relationships as defined by the person is a foundation to enable recovery. In this way, recovery involves dynamics between the person and her/his contexts [9,11]. Respect for personal choices and self-determination are considered core values, thus recognizing the person as an expert and lived experience as valid knowledge. However, subjective experiences of what recovery and improved quality of life involve may not align with the more outcome-oriented clinical perspective of recovery [6,12].

Understanding recovery as encompassing personal, social, and contextual processes is related to the historical roots of recovery that can be traced back to the civil rights movements that began in the 1960s. This understanding is based on ideas of empowerment, self-help, and human rights, and emerged as a response to suppression, stigma, and paternalistic services in mental health and substance abuse [11,13,14]. The early recovery movement took place outside mainstream services and was at heart a social justice movement and a protest against power imbalances between health professionals and service users. In this way, it touched upon epistemic questions related to the determination of what valid knowledge is and who should be recognized as “knowers.” The importance of acknowledging people’s lived experiences of how they navigated through mental distress and lived meaningful lives is emphasized, recognizing knowledge as subjective and contextual [11,15]. Due to its subjective and contextual nature, recovery is not a stable and coherent state of being, and experiences of recovery change over time and in relation to context [6,15]. In this regard, it may be pertinent to question whether the past decades’ recovery orientation of services has led to a professionalization and standardization of the concept of recovery, shifting the understanding of recovery and its values and knowledge base in a more streamlined and clinically and professionally led direction [16].

In Norway, which was the main site of the studies included in this meta-synthesis, recovery-oriented practices and perspectives have been emphasized as desirable in national guidelines. The importance of understanding and recognizing people struggling with mental health and substance abuse issues as citizens and part of a community is underlined. This involves a recognition of the right to live a life of dignity in the community and to receive local and helpful support that is attentive to the person’s needs and wishes [17,18]. Some Norwegian municipalities have mandated that their mental health and addiction services should be recovery-oriented. To date, there are no specific national programs based on recovery, thus understandings and practices of what recovery and recovery orientation involve remain diverse [1,17]. However, in understanding recovery as social and contextual processes, local variations focusing on context and local culture may be perceived as natural and desirable.

In a social and contextual understanding of recovery, emphasis is placed on exploring the multifaceted consequences of mental health and substance abuse issues on people’s everyday lives and the myriad of ways in which people deal with these, rather than on unambiguous definitions. This also includes how social conditions, inequity, and marginalization related to such issues affect people’s lives and possibilities [19,20,21]. With its subjective, collaborative, and relational nature, recovery can be understood as a dynamic and open concept that cannot be “implemented” in services [15,22,23]. More specifically, in a research context, this could be perceived as shifting the focus from aiming to define what recovery *is* to describing and exploring how it can be understood in terms of personal and social contexts.

This paper is the first part of a tripartite study addressing the following research questions (RQ): (a) what are the characteristics of experiences of recovery? (b) What are the characteristics of processes of recovery? and (c) What are the nature of and experiences with recovery-oriented mental health and substance abuse services? In addressing these research questions, we have carried out meta-syntheses of empirical papers published by the research group of the Center for Mental Health and Substance Abuse (CMHSA) at the University of South-Eastern Norway. The findings will be presented in three separate papers, focusing on one research question in each part: Part 1, which is this paper, addresses experiences of recovery (RQ 1), Part 2 focuses on processes of recovery (RQ2), and Part 3 deals with recovery-oriented practice (RQ3).

## 2. Method

### 2.1. The Research Context

At the CMHSA, recovery has been a key area of research since the early 2000s. The center has a specific focus on collaborative research methodologies with people with lived experience, family members, and practitioners. The CMHSA engages people with a variety of experiences and a wide range of knowledge as key partners in research. Our recovery research has from the outset focused on subjective experiences, relational aspects, everyday life experiences, and the impact of material and social conditions as well as recovery-oriented services, community-based support systems, and peer support work. Furthermore, the center conducts research on dialogical and collaborative practices and child and adolescent issues. The researchers have varied professional backgrounds in the health and social care sector and a wide range of clinical practice experiences in addition to lived experience. The center has expertise in qualitative, quantitative, and triangulation/mixed methodologies.

### 2.2. Qualitative Meta-Syntheses

The method applied in this study is a form of qualitative meta-synthesis. The term qualitative meta-synthesis has various meanings, refers to a variety of approaches, and is often used in systematic review studies. The qualitative meta-synthesis in this study is in line with the first kind of synthesis identified by Sandelowski, Docherty, and Emden, which referred to integrating the findings from multiple qualitative studies within a program of research by the same investigators [24]. The purpose of this approach in the present study is to explore how recovery is described in empirical research at the CMHSA, addressing the research question of “How is recovery described in empirical research at the CMHSA in the period 2005–2020?” The objective is to arrive at a theoretically meaningful synthesis about recovery as experiences, processes, and service orientations through the integration and comparison of the qualitative empirical material accumulated by CMHSA researchers in their studies of community mental health and substance abuse practices. The procedural steps adopted reflect the seven steps identified by Noblit and Hare for meta-ethnography, which consist of (1) getting started, (2) deciding what is relevant to the initial interest, (3) reading the studies, (4) determining how the studies are related, (5) translating the studies into one another, (6) synthesizing translations, and (7) expressing the synthesis [25].

The publications included in this meta-synthesis were written by CMHSA researchers, whose research orientation as a group is recovery and recovery-oriented practice. The focus of our synthesis was recovery experiences, processes of recovery, and recovery-oriented mental health and substance abuse practices. The first four steps of Noblit and Hare’s method have been well established within the group. Thus, this qualitative meta-synthesis encompasses the last three steps, namely translating the studies into one another, synthesizing those translations, and expressing the synthesis. Meta-ethnography and meta-syntheses in general are oriented to “synthesizing” researchers’ interpretations of qualitative data in original studies, which are social constructions “built into accounts of methods, in the theories used, in the researchers’ worldviews” ([25], p. 3). However, this meta-synthesis did not have to deal with the issue of consolidating different perspectives or worldviews. It began with the prior knowledge of our perspectives, methods, and worldviews, which align with the epistemological stance of a phenomenological–interpretive and critical perspective.

For the fifth step of translating the studies into one another, the themes and concepts from each study with their descriptors were identified, compared, and contrasted, which also involved reflections on relevant literature. Based on the results from the fifth step, the sixth step involved meta-synthesizing the themes and concepts regarding recovery experiences, processes, and practice orientations. Thus, this step involved using the researchers’ judgment and creativity, which is critical in qualitative synthesis [26]. The synthesis of themes and concepts found in these publications involved consolidating similar themes and specifying them into meta-themes by comparing the themes and their meanings. Some themes extracted from individual publications were also specified as meta-themes when considered critical in providing the meanings of recovery experiences, processes, or practice orientations. The seventh step of the meta-synthesis, “expressing the synthesis,” involved systematizing the results of the meta-synthesis.

**Figure 1:**
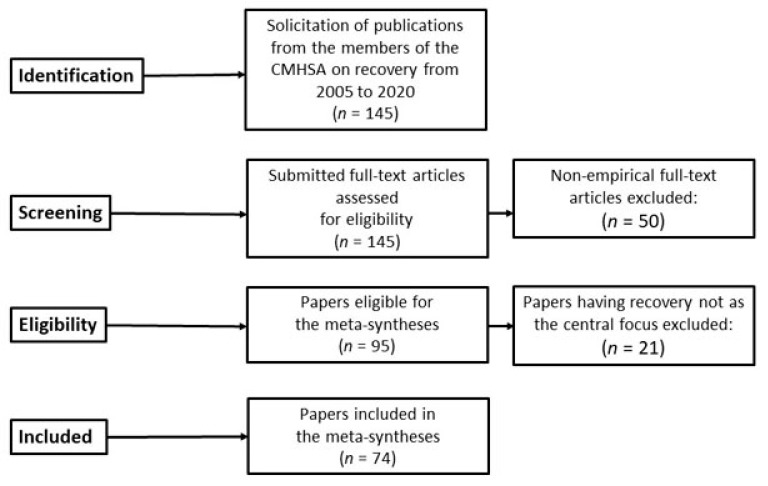
Flow chart of the process of gathering the publications for the meta-syntheses.

Figure 1 shows the steps taken by the research team for the meta-syntheses for Parts 1, 2, and 3 using a PRISMA flow diagram. The details of the steps followed in assembling the database for this study are somewhat simplified because the publications included in these meta-syntheses were those of the members of the CMHSA research team.

The steps of collecting, reviewing, and analyzing the papers were as follows: a core research group of five CMHSA researchers was established to be responsible for the meta-syntheses and writing the results for publication. All 20 researchers in CMHSA were then invited to contribute to the study and requested to submit their publications to the core group. Sixteen researchers accepted the invitation. The inclusion criteria for the publications were empirical papers published from 2005 to 2020 with a focus on recovery as personal, social, and relational experiences and processes and on recovery-oriented services. We also invited the researchers to include other papers that might be relevant to the topic. The languages included were English and Scandinavian languages (Norwegian, Danish, and Swedish). A total of 145 papers were submitted.

These papers were reviewed by the core research group in relation to the research questions, resulting in the final selection of 95 empirically oriented papers. Each of these papers was systematized by using a data extraction form inspired by the Critical Appraisal Skills Program (CASP) for quality appraisal in qualitative evidence synthesis [27]. These studies employed qualitative methods, mostly focus group and in-depth individual interviews with research participants who were service users, family members or significant others of service users, and professionals. The analytical methods used in these studies were descriptive and/or interpretive.

An examination of this set of publications by the core group resulted in a division of the material into three broad topic areas: (a) recovery as personal and/or contextual experiences, (b) recovery as processual, and (c) recovery-oriented services and practice. Therefore, three meta-syntheses were performed using these data. There were 28 papers in the topic areas of recovery as person–context experiences and as processual, and 46 papers in the topic area of recovery-oriented services and practices. We planned to write three papers, each focusing on a meta-synthesis of one of the three topic areas. The 28 papers mentioned above formed the basis of two meta-syntheses presented in the papers as Part 1 and Part 2. This paper deals with the meta-synthesis of recovery as personal and/or contextual experiences.

## 3. Results

This meta-synthesis presents results regarding experiences of recovery as personal/contextual, while results regarding processes of recovery will be presented in Part 2. The meta-syntheses for these two parts were on the papers listed in Table 1.

The included papers are listed chronologically in Table 1. The table includes brief descriptions of methods used, research participants, and important themes describing and exploring dimensions of recovery.

The studies were conducted in the context of community mental health and substance abuse practice. They included participants with experience of diverse mental health and substance abuse difficulties, both acute and long-term, who had received a variety of mental health and substance abuse services. Six of the studies were based on a multi-national collaboration between Italy, the USA, Sweden, and Norway. The other twenty-two studies were based solely in a Norwegian context.

The meta-themes in this presentation are based on a consolidation of similar themes in the included papers, followed by a synthesis of the themes and their meanings into overarching meta-themes across the set of included studies. The synthesis aims to capture overarching patterns and themes, but also variety and diversity as important factors in how recovery as person–context experiences and dynamics is described.

Recovery as person–context experiences and dynamics encompasses descriptions of recovery as involving complex dynamics between personal experiences of what recovery may involve entangled with contextual dimensions such as relationships and networks, social settings, attitudes and values in society, and access to material resources.

Our analysis of the data revealed five meta-themes that are further described below: (a) being normal, (b) respecting and accepting oneself, (c) being in control, (d) recovery as intentional, and (e) recovery as material and social.

a Being normal

The term “being normal” refers to being able to carry out ordinary everyday activities, belonging in social settings as an ordinary person, and participating in social life as an ordinary social agent fulfilling common obligations.

Normality/“being normal” is oriented towards how recovery unfolds within the context of normal activities and environments. “Normal,” in this context, is connected to the idea of which settings and activities the majority of people are involved in. Experiences of being normal seem to be closely connected to the possibility to participate in such activities, settings, and roles “just like anybody else.” Participation in ordinary everyday activities, being in ordinary social settings, and the possibility to fulfill typical social roles and obligations are all described as crucial elements of being normal [30,34,35]. 

The concept of everyday activities covers a wide range of activities and routines, such as housework like cleaning and cooking, and a variety of leisure and work-related activities. Taking part in these activities may provide structure and regularity in everyday life. It also enables connections with ordinary settings and people that can provide a break from psychiatric settings and from the role of being “mentally ill” [30,35,39]. Ordinary social settings are referred to as being recovery nurturing through providing people with the possibility of doing normal activities in the same settings as others, having normal conversations, and being able to step out of the patient role [30,32,35]. Furthermore, being and participating in ordinary settings comes with certain expectations and obligations such as being on time, doing what one is expected to do, and being in situations of giving and receiving. Being met with expectations is part of being normal and allows for a role as a person with certain skills and capacities who can also be relied on [34,39].

Participation in normal settings and contexts in the community and in everyday family life can pave the way for an interplay between personal processes, being with others, and doing things that feel meaningful, thus promoting a sense of normality and of being part of society [30]. Experiences of being normal are highly dependent on access to environments and activities that are considered parts of ordinary everyday life; therefore, participation in activities in the context of mental health facilities does not hold the same potential [34].

b Respecting and accepting oneself

Respecting and accepting oneself encompasses personal and relational dynamics related to the past, present, and future that are crucial to recovery. This refers to multidimensional aspects of being oneself from the perspective of the self as well as in terms of validation by others.

An essential idea in recovery is to emerge as a person in terms of coming to accept and appreciate oneself and daring to come forward “as one is” [46]. This relates to working towards becoming who one wants to be in the near and distant future, and also to become oneself again, in terms of retrieving some of what seemed lost due to mental health problems. The processes of becoming and retrieving require hard work in terms of gaining insights and struggling to achieve something [7,30,46]. Engaging in this hard work and proving to oneself and others that one is capable and does one’s best is important in learning to love oneself. Being involved in these processes also enables new understandings of one’s past self and of future possibilities, i.e., understandings of who one is and of how one’s potential can change, both in the eyes of oneself and others. In this way, these processes can enable one to accept and love oneself. Support from family and friends can be important in loving and accepting oneself. For others, spiritual support (for example, from a God), being in nature, or having a pet can fulfill this need [7,46,54]. 

How one is regarded and met by others is crucial in gaining respect and accepting oneself, and this typically involves being seen as more than one’s diagnosis [34,35,49]. The possibility to participate and interact with others in work-related and everyday activities is an important prerequisite for moving beyond diagnosis and gaining self-acceptance [7,30,39,46]. Through such interactions, one becomes a person who contributes and has something to offer to others. Being valued as something more than a psychiatric patient appears to be crucial to self-acceptance and recovery.

c Being in control

Being in control refers to the importance of gradually taking the lead in mastering one’s life, finding ways of caring for oneself, and knowing one’s possibilities, resources, and signs of ups and downs. Taking control is not easily done once and for all; on the contrary, it requires continuous practice in how to master life. Life mastery is a skill that can be developed through everyday practices, where the ability to try out and gain new experiences in diverse settings and activities is crucial. The support of others and access to supportive environments is important in this regard, and other people can both facilitate and hinder these processes [36,39,46]. Being aware of one’s signs of ups and downs allows one to take necessary precautions in order to live with and deal with mental distress and to be in control. Insight into these signs also enables the person to develop ways of giving meaning to these experiences. To many, this is a requirement for positive change in their lives [38]. Such a focus could also involve support from significant others and the ability to take feedback from them [7,38]. 

While mastery can be related to acquiring new experiences, insights, and skills, it is also important to balance this with taking care of oneself and knowing where one’s limits are. Recognizing and taking signals from oneself and others is important in this regard, and involves being attentive to bodily, emotional, or social signals. The ability to recognize these signals and to find ways to rest and recuperate is important in taking care of oneself [35,45,54]. While being attentive to signals is vital to being in control, knowing one’s possibilities and resources is also of great significance. Getting to know or rediscovering one’s abilities and what activities and relations give energy, joy, and a sense of mastery is vital, and these are both personal and relational processes [38,45,46,54]. Thus, being in control appears to revolve around the dynamics of challenging oneself or being challenged balanced with the necessity for withdrawal and respite, understood as personal and social processes.

d Recovery as intentional

Understanding recovery as intentional refers to dynamics and actions, with or without support from others, that people intentionally engage in to get better. This involves dynamics between self-defining aspects of who one “is” through learning to accept oneself, being good to oneself, and establishing a degree of self-control and coping strategies on the one hand, while being open to change and developing a capacity for change on the other hand. Issues connected to stability, acceptance, and efforts to change appear to be entangled, presupposing one another.

Embarking on a recovery journey rests upon a determination to accept *and* change various issues in life in order to live the best life one can. A determination to get better can be expressed through doing something concrete to improve one’s situation, or “just doing it” in terms of being creative in developing coping strategies in dealing with difficulties and finding solutions for what works [7,34,37]. Others’ help, support, and belief in one are important in this regard [31,36,37,52]. However, finding the courage to engage in processes and actions to work towards small and larger goals in life is also determined and supported by learning to accept oneself and finding ways to care for oneself. This also rests upon gaining insight into conditions and relations that support or hinder recovery, taking control of how to deal with these issues, and making positive choices for oneself [51,52]. It involves holding on to one’s goals and dreams while also being open to changes and actively and dynamically taking part in environments and contexts that can support changes [38,42]. Part of this is dynamics between being and becoming and between accepting and striving for change, in relation to contextual factors [32,42]. This can involve insight into what to accept and hold on to, in a dynamic relationship with questioning and changing one’s habits, attitudes, and values and making choices on being prepared to participate in the mainstream or not, with the possibilities and limitations this may involve [43,47,51].

e Recovery as material and social

This theme refers to how recovery is understood as dynamic processes between the person and relational/material contexts. This includes having a social life and engaging in activities, involvement across various community settings, performing the role of a citizen in society, having a job, and the importance of having a house and a home.

In addition to one’s own efforts and persistence, one of the elements that makes recovery possible is having access to social life and engaging in activities with others. Contact with others can facilitate access to recovery-promoting tasks and settings such as activities of daily living and leisure activities, thus supporting the person’s sense of being someone outside the mental illness setting [30,35]. Having a job can also be important for developing a social life and social skills [7,30,32,35,39]. A positive social life enables joy and fun, providing valuable respite from the hard work of being in recovery, and involves inclusion in broader society [31,32]. Fulfilling a social role and participating in social settings are crucial to regaining a life and status as someone who takes part in society, not “just a mental health patient.” Being able to contribute to society and not merely being a passive care recipient is essential for the sense of being part of a community and a valuable citizen [46]. Thus, the dynamics between the person and recovery-promoting contexts can enhance participation and citizenship. On the other hand, lack of access to social and material resources can be a barrier to participation and recovery [32,34,36]. 

Recovery is promoted by necessary material conditions such as satisfactory housing and personal finances [28,34,36]. Adequate material resources such as having a sufficient income are connected to the person’s possibilities to participate and contribute in society and its activities and to feel valued as a person [28]. Having a home is paramount to recovery, which underlines that a home is something more than just shelter. It is a place for privacy, safety, and retreat, and can also enable social activity [29,46,53]. The material context of a home interplays in dynamic processes with personal development in that the person deals with everyday tasks and finds hope and confidence. Home also serves as a safe place for developing coping strategies as well as enjoying hobbies and being with others [29,36,53]. 

## 4. Discussion

This paper synthesizes the characteristics of experiences of recovery. The meta-synthesis shows how deeply personal experiences are entangled with contextual dimensions such as relationships and networks, social settings, attitudes and values in society, and access to material resources. The interrelatedness of the personal and contextual dimensions can be perceived as dynamics, in terms of being subject to continuous change and mutual interaction. By synthesizing these experiences of recovery as dynamics, we suggest an understanding of recovery as an on-going interplay between the person and others, and between the person and material resources.

The discussion will reflect on two important, overarching dynamics: (1) recovery as dynamics between the self and others and (2) recovery as dynamics between the self and material contexts.

### 4.1. Recovery as Dynamics between the Self and Others

Our analysis suggests an understanding of recovery as dynamics involving the individual and others. These dynamics can be understood in at least two ways. On the one hand, the person develops a way of *being* through providing stability to life, by accepting, being good to, and controlling her/himself. On the other hand, the person is also *becoming* through striving towards change and a “normal life.” Being in recovery is thus about the dynamics of the parallel processes of being/stabilizing and becoming/changing. The four themes that emerged in this analysis (i.e., being normal, respecting and accepting oneself, being in control, and recovery as intentional) suggest the critical importance of the self in recovery in this overarching dynamic. This involves processes of the self as striving in, moving with, and handling one’s living, but at the same time the critical role of others in terms of the dynamics in play that involve the self and others, which can be related to and worked on, relied on, and managed in social settings. This means that while the locus of recovery is in the person, recovery experiences involve the dynamics of the self and others in social situations.

Price-Robertson, Obradovic, and Morgan argue that recovery is a social and relational process based on the idea that human beings are interdependent creatures [23]. Experiences emerge at the intersection between people, their relationships and their environment; from a relational perspective of recovery, there is an inseparable interdependence between the individual and the community. In our material, the twofold dynamics of recovery as a constant shift between being/stabilizing and becoming/changing play out in a context where person and community interact. There is also an interdependent relationship between being/stabilizing and becoming/changing because one does not exist without the other. The shifting between the two positions takes place for the individual in her/his environment and can thus be understood as a dynamic between self and others.

Our findings synthesize descriptions of recovery that seem to draw on an individual, or personal, view of recovery as things the person does, such as *respecting and accepting oneself* and *being in control*. Yet, the descriptions include the importance of others. In accordance with the concept of relational recovery, persons and experiences are understood contextually. What a person does will also include the person’s relationally developed resources. This is emphasized in recovery narratives; here, although the individual has power as the author of her/his own recovery narrative, co-authors are included in the narrative. We relate to and “talk” with “others” from our past and with people, social structures, and culture in the present [55]. Relationally created resources from the past can create present opportunities and future expectations, and concrete present resources [56]. Personal recovery narratives are polyphonic in the sense that the self is not the only protagonist. Considering recovery as relational dynamics entails illuminating conditions in the environment that are part of these dynamics [57].

Price-Robertson et al. describe what they refer to as “… *relational recovery*, which is a way of conceiving recovery based on the idea that human beings are interdependent creatures; that people’s lives and experiences cannot be separated from the social contexts in which they are embedded” ([23], p. 109). Recognizing relationships as part of recovery is insufficient, since recovery is entirely pervaded by relationships. Phenomena such as hope are simply inconceivable outside contexts [23]. Duff claims that if contexts are ignored, one risks perceiving recovery as “… a given individual’s *effort or will to recover*” ([58], p. 62).

Drawing on the philosophy of Gilles Deleuze, Roberts argues that the subject should not be understood as some innate, stable entity located within the individual [59]. According to Roberts (2006), Deleuze’s work “… suggests to contemporary mental health care that, rather than being ‘stable’ or ‘fixed’, subjectivity should be understood as ‘dynamic’ and in a continual state of ‘evolution’” ([59],(p. 199). In regard to our findings, this seems to harmonize with the dynamics between the person and the self, and the self and others. Trying to accept oneself goes hand in hand with the need to be accepted by others and fit in.

Price-Robertson et al. argue that the opposite of individualism is not collectivism, but interdependence [23]. This interdependence underlies ecological thinking, where people are perceived as inseparable from their environment. Furthermore, it acknowledges the needs of both the individual and the collective. People are obviously dependent on how others respond and relate to them [60]. A person’s past experiences may include a lack of recognition from specific others and society. This lack of recognition is not necessarily acceptable as part of oneself [61]. When experiences are understood in light of oppression and discrimination and not as personal faults and shortcomings, this can lead to changes in the experiences of the present and what one would expect to experience in the future [56]. Finding a way to dissolve the ontological connection between identity and mental health (problems) would be one way of combatting stigma [58].

Duff links both human and nonhuman spaces, along with bodies, objects, and forces, as affecting the individual in recovery and others in “… an always unfinished process of combatting the stigma of mental illness and its ‘spoiled identities’, managing the symptoms of illness and their personal and social effects, rebuilding social networks, and maintaining a sense of hope for the future. All of these things are lived and felt” ([58], p. 66). The idea of co-existence (*both/and*) seems to be more prevalent in our findings than that of “one” (*either/or*). For instance, holding on to what one has and what one is seems to go hand in hand with constant change and becoming. Furthermore, searching for a place to thrive and to fit in goes side by side with accepting oneself “as one is.” It seems futile to see these aspects of recovery as separate from one another.

In the recovery literature, there is a tension between emphasizing personal change processes and creating recovery-nurturing environments [62]. A central aspect in our findings is *being normal*, in the sense of being able to participate in “normal” activities. Relational recovery means that in order to accept oneself, one must be accepted by others. Not participating in “normal” activities such as employment may imply non-acceptance by others, making it difficult to accept oneself. At the same time, it can be necessary to accept oneself and develop a strong self in order to gain access to and participate in “normal” activities [63]. Price-Robertson et al. argue that the interdependence between the individual and the environment is the kind of interdependence that underpins system and ecological thinking [23]. Gregory Bateson argues for a cybernetic understanding of all systems: the individual organism, social systems, and ecological systems [64]. All systems have a thermostat that regulates stability and change. Recovery understood as a dynamic between self and others means an effort to include both the individual as a self-regulating system and the broader system around the individual. “Being normal” can be understood as the thermostat in the self-system for the person in recovery, with the intention of getting better, through becoming what may be considered an ordinary person, doing ordinary activities in an ordinary life, and being an ordinary social agent fulfilling common obligations. Being in recovery can thus be understood as striving for equilibrium between being/stabilizing and becoming/changing, in a thermostatic self-system. In this self-system, “being normal” should not be understood as normative, but as a thermostatic regulation point for creating the equilibrium when the person wants to leave a more “unnormal” and unordinary life. In this sense, Price-Robertson et al. also underline that disconnecting from relationships and setting boundaries are interpersonal acts [23]. An essential factor seems to be the dynamic and tension between how much the person should change in order to adapt to norms and demands and how much others should change in order to accept the person.

### 4.2. Recovery as Dynamics between the Self and Material Contexts

The findings of this study also highlight how recovery can be perceived as dynamics between the person and material contexts, suggesting the need to conceptualize a person’s recovery in interplay with the person’s possibilities to have a safe home, along with access to work and activities, money, community settings, and services. The study underscores how mental health and substance abuse issues need to be considered as both personal and contextual, where both form part of an ongoing, dynamic relationship [15]. Thus, deficient material resources are not to be understood as personal problems, by assuming that mental health disorders cause unemployment, poverty, and housing difficulties. On the contrary, it may be that material and contextual issues are causing and/or intensifying mental distress [21,29,36]. Understanding recovery as dynamics between the person and material contexts suggests that contextual factors influence *and* are influenced by the person. Thus, given this dynamic, neither the person nor contexts are to be perceived as stable concepts. In addition to *being*, these issues are also mutually *becoming* through dynamic interactions.

Material contexts are viewed as necessary elements for people in general in their existence, belonging, and functioning and they are also crucial to recovery in terms of providing settings and resources that enable experiences of meaning, connection, and participation in the community [65,66]. However, people living and dealing with mental health issues often experience challenges in gaining access to housing, employment, and various public and social arenas [65]. While the lack of access to material settings and contexts may serve as a direct obstacle to recovery, in line with the findings of this study, we also suggest that the inability of people to contribute to and influence their material contexts is contrary to recovery. Constantly being at the receiving end of relations to other persons or material systems represents a power imbalance and a possible sense of being dependent and not in control [34]. Thus, recovery encompasses feeling useful and being able to contribute to and influence material contexts, in a dynamic relationship with personal development. Since people in recovery are likely to be disadvantaged in terms of material context and resources, their efforts at recovery in this area are often influenced by the contextual dynamics that exist in social systems. In this sense, access to basic material contexts and resources is linked to fundamental human rights, thus recovery can very well be placed within a rights-based understanding of mental health [67]. Davidson et al. emphasize the importance of persons in recovery having the same rights as other citizens, including the right to decide where to live [11]. However, persons in recovery are often unable to compete in the housing market on equal terms with the general population [68,69]. Instead, their municipality provides them with supported housing [70]. Supported housing allocated to persons in recovery may not convey the feeling of a home to those people, underscoring how a house does not necessarily equal a home. While a house may serve the purpose of providing shelter, a home is also a place for privacy, safety, retreat, and social activities. Thus, following Doroud et al., a home is a place for being and doing, and can also be a place for becoming, in terms of fostering hope for a better future and recovery [65]. Safe places or atmospheres, sometimes found within a home, seem to be a prerequisite for identity work. A safe place to live and having personal belongings form part of an assemblage that facilitates the process of recovery [58].

Not having the resources to choose accommodation and a community that meets one’s preferences and needs may result in a lack of sense of home, with all its possible implications [29,53]. The current study shows the potential influence of a person’s community and material contexts on experiences of recovery. It emphasizes the need to develop measures that will help persons in recovery to gain adequate social and material resources. This will in turn support their ability to exercise their *rights*, and to participate with others in the housing market and in other potential recovery-promoting settings.

Having a safe and secure home, sound finances, and other fundamental material resources and capacities fosters recovery. Furthermore, as shown in this study, employment gives meaning and a sense of mastery, identity, and self-esteem, which are all factors that promote recovery by making it easier to perform recovery-enhancing activities. They also strengthen the possibility of having recovery-promoting social contacts and giving and receiving social support.

There is broad agreement that societies which give all citizens the opportunity to play a full and useful role in economic, cultural, and social life will demonstrate better mental health than societies where people experience exclusion, alienation, and insecurity [71,72,73]. Despite having a high standard of living, low unemployment, and good welfare schemes, there are huge social and civil health inequalities in Norway [34]. Barstad stresses that “one of the most important goals of the welfare state is to provide economic security in case of sickness and disability” ([74], p. 5). Findings from the current study indicate that the Norwegian welfare state struggles to reach this goal. In general, persons with mental health and/or substance abuse problems often report lacking an inclusive working life, higher education, and financial security [21]. Duff points out that the neo-liberal political flow is shifting the responsibility for the management of mental health problems from the state to civil society and the individual. Duff argues that atmospheres, environments, and contexts play an important role in recovery and that the state has a crucial responsibility in this regard [58]. The findings in the current study point out that structural factors related to education, employment, and finances influence recovery through ongoing dynamics with the person. With an emphasis on the importance of material contexts in recovery, the study underlines that recovery is not only an individual or social matter, but also a political responsibility. Henwood and Whitley point to the increasing focus on what constitutes recovery-oriented health services, while less attention has been paid to what would constitute a recovery-oriented society [75]. In view of the findings in this study, we argue that a community response is vital to improve material contexts that may serve as recovery-enhancing aspects of person–context dynamics.

This study underlines how recovery is deeply interwoven with material resources. This corresponds with the emphasis of the UN Special Rapporteur on the need to recognize that health improvements require measures on the societal level and are linked to social determinants [67]. However, the findings also suggest that through ongoing interaction with material resources and the opportunity to influence them, even on a small scale, the person may develop a sense of contributing, being useful, and being in control.

The two overarching dynamics encompassing the five meta-themes elicited from this meta-synthesis can be viewed as representing the essential, experiential characteristics of recovery. They elaborate on how persons with mental health and substance abuse problems make efforts to have fulfilling and satisfactory everyday lives within the constraints of the difficulties, vulnerabilities, and vicissitudes imposed by their problems.

Our findings align with the results of a thematic synthesis by Dell et al. of 25 systematic reviews on recovery within an ecological framework. The themes generated in that study were (a) recovery as a process of overcoming despair to realize a positive sense of self and well-being, (b) environmental requirements necessary for recovery, (c) the role of autonomy, control, and personal responsibility, (d) the importance of social support and meaningful activities to the development of a sense of belonging and purpose, and (e) developing acceptance of one’s illness and insight into how to establish and maintain wellness ([76], pp. 7–8). The general tenet of these themes emphasizes the interplay between the self and the environment, which is the key feature of the five themes in our meta-synthesis. With their focus on the characteristics of experiences of recovery, the five themes coalesced into two overarching dynamics in our study, emphasizing the critical features of recovery experiences expressed in terms of the relations of the self with others and the role of material/contextual resources. This positions the findings of our meta-synthesis within a social understanding of recovery as described and explored in a critical commentary paper by Ramon, based on fifty-nine studies [77]. The paper highlights the centrality of looking at recovery as simultaneously consisting of social *and* personal dimensions, suggesting that in understanding and responding to people’s mental health difficulties, it is vital to include the social context. Our meta-synthesis underscores how material, social, and personal elements of recovery interact in dynamic and ongoing relationships, where one of these cannot be separated and understood except through its relationship with the others.

Furthermore, our study results also emphasize experiences of recovery as a depiction of human living in the context of mental health and substance abuse. Kim identified four dimensions of human living in terms of (a) living of one’s body, (b) living of oneself, (c) living with others, and (d) living in situations [78]. The two dynamics of the recovery experiences specified in our study emphasize this notion in terms of the integrative and interdependent nature of human living. Recovery as a form of human living involves the living of the body and the self in integration with living with others and in situations in terms of being normal, respecting and accepting oneself, being in control, being intentional, and consolidating material/situational resources.

### 4.3. Strengths and Limitations

Although this meta-synthesis of the studies by one research group (the CMHSA) has strengths in terms of its findings being coherent and integral, framed by the perspective of the research team, it also presents limitations because of the study’s orientation to a specific perspective. The findings are limited by how the data were analyzed in the original studies and by the fact that this meta-synthesis was conducted from an interpretive perspective. It is possible that a more comprehensive, diversified understanding could have been gained by a meta-synthesis of studies with a greater variety of perspectives and analytic methods. However, the richness of the findings in the study adds to our knowledge of the experiences of recovery, providing in-depth understandings gained from an analysis that took into account the perspectives of the research participants. A related limitation concerns the generalizability of the findings in characterizing experiences of recovery since the studies were mostly carried out in Scandinavian countries. However, if we generally accept the idea that experiences of recovery are universal human experiences in the context of mental health and/or substance abuse problems, the findings enrich our understanding of the phenomenon of recovery.

## 5. Conclusions

The results of this meta-synthesis provide a consolidated picture of the core features of experiences of recovery as the dynamics of the self and others and the dynamics of the self and material resources. These results suggest that as recovery experiences are embedded in the interdependence and interplay between the self and the social/material environment, it is critical to view recovery as a process rather than an outcome [12]. Recovery is a process of human living in the context of difficulties and limitations imposed by mental health and substance abuse problems. Thus, the emphasis has to be on “becoming,” “changing,” and “discovering” rather than achieving outcomes. These findings have implications for mental health and substance abuse practice in terms of indicating the kinds of support that are critical to helping service users in their recovery experiences. In line with an understanding of recovery as person–context dynamics, the findings suggest the need to work not only with the person, but also with families, networks, social systems, and local communities. Furthermore, this understanding acknowledges how the person is not only influenced by context, but also how she/he actively contributes to the context. Understanding recovery as person–context dynamics supports the understanding that recovery is not a stable and context-independent concept that can be implemented in services. We suggest that a dynamic understanding of recovery can inspire mental health and substance abuse services to develop in more collaborative, open-ended, and context-sensitive directions.

## Figures and Tables

**Table 1:** List of the empirical papers included in the meta-synthesis (in chronological order of publication).

Publications	Research Question(s)	Methods	Research Participants	Themes and Meanings
Davidson, L. et al. [7]	Explore processes of recovery in psychosis	Narrative and phenomenological approach with individual interviews	Twelve adults with experiences of recovery in psychosis	The person’s determination to get better, establishing a degree of self-control, and struggling to achieve a normal life in dealing with problems The need for material resources and a sense of home, and the importance of going out and engaging in normal activities Roles of formal/informal health systems in terms of the benefits of medication, involvement in mutual support/user groups, and participation in various psychosocial interventions The need to be accepted and to accept oneself as a normal person who exists beyond the psychosis; the impact of stigma and discrimination, and the importance of having one’s rights respected and returning to a meaningful social role through work and/or positive relationships outside of the formal mental health system The roles of social and cultural factors for the persons in terms of opportunities and support offered
Sells, D. et al. [28]	To describe service system contexts in which the informants lived and received services and support	Phenomenological narrative interviews	Twelve persons in recovery	Roles of home, significant others, and coping strategies being interwoven in the context of individuals’ lives and personal recovery journeys
Borg, M. et al. [29]	How do people in recovery from psychosis develop and accept their role in society and where does that take place?	Qualitative interviews	Twelve adult service users in recovery from psychosis	Material resources in terms of their practical importance in daily life and their immaterial meanings, such as emotional comfort Having a home, meaning having a place for growth and development, a place of control, an opportunity to balance privacy and social life, and a place to long for and dream about
Sells, D. et al. [30]	To identify community settings that appear to foster recovery, as well as the mechanisms through which this takes place	Qualitative individual interviews	Persons in recovery from psychosis	Involvement across various community settings can establish more beneficial and lasting understandings of the self Being understood and accepted Fun and enjoyment Role shifting Meaningful routines Employment Spirituality Esteem Anger as a mechanism of empowerment and change Integrative aspects
Topor, A. et al. [31]	(A) Can other people contribute to the recovery process? (B) If so, which people? (C) According to the informants, what do these people do that contributes to the recovery process?	Qualitative interviews	Twelve persons in recovery	Social relationships play key positive and negative roles in recovery processes A beneficial relationship is not dependent on the helper’s formal education or training Beneficial relationships are characterized by: (a) standing by the person with continuity, (b) being bearers of hope, (c) demonstrating through being there that the person is more than his/her illness, and (d) being there for the person in recovery, including providing practical support, intervening as advocates and lobbyists Key characteristics of helping in recovery processes: (a) being there for the person in recovery, (b) helping by doing more than expected, and (c) helping by doing something different than what was expected
Mezzina, R. et al. [32].	How do people in recovery from psychosis develop and accept their roles in society and where does that take place?	Qualitative interviews	Persons in recovery from psychosis	Social barriers to recovery: Stigma (and self-stigma) Being different (labeled) Exclusion and stigma (from normal social life, locked into role of mental patient) Social pathways to recovery: Self-advocacy Being in supportive social environment Finding new bonds and new roles Working and studying, thus enabling new roles and statuses Participation and citizenship with a sense of belonging
Biong, S. et al. [33]	How is meaning constructed in narratives of suicidal behavior?	Phenomenological hermeneutic approach with narrative interviews	Four adult males receiving substance abuse services	The meaning of living with suicidal behavior as a movement between different positions of wanting death as an escape from pain and hope for a better life: The meaning of relating The meaning of reflecting The meaning of acting
Borg, M. et al. [34]	To explore recovery within the context of the person’s everyday life	In-depth individual interviews	Thirteen adults in recovery	Being normal Just doing it Making life easier Being good to yourself
Borg, M. et al. [35]	To identify and discuss the role that work plays on the road to recovery for people with severe mental illness, particularly those diagnosed with psychosis	Phenomenological approach with in-depth individual interviews	Thirteen adult users with mental health problems	Being and becoming: an active worker not a passive patient Belonging in an ordinary working life Balancing—not too much, not too little Believing in oneself—the importance of supportive and flexible environments
Topor, A. et al. [36]	To broaden the individual perspective on recovery by describing additional aspects of the journey that involve the contribution of others and various social factors and elements that can facilitate or impede inclusion in community life	Qualitative individual interviews		The contribution of others, including friendship, families, and professionals Social factors including home, money, and employment Structural recovery, including the need for recovery knowledge, including recovery of others and recovery of the services
Herrestad, H. et al. [37]	How meaning is constructed in narratives of hope by persons that have recently engaged in suicidal behavior	Hermeneutic–phenomenological approach using semi-structured in-depth interviews	Twelve adult patients admitted for overdose of medication	Relational hopes for life and death The meaning of hope for life—hope in the context of relationships The meaning of the act of hoping projected as definite or indefinite hopes in terms of “stop or not,” “a limit or not,” and “a specific agency or not”
Veseth, M. et al. [38]	What do individuals with bipolar disorder do to promote their own recovery and what challenges do they meet?	Hermeneutic–phenomenological approach with individual in-depth interviews	Thirteen persons with bipolar disorder	Handling ambivalence about letting go (i.e., accepting) of manic states Finding something to hang on to when the world is spinning around Becoming aware of signals from self and others Finding ways of caring for oneself
Borg, M. et al. [39]	To understand the role of work in recovery from bipolar disorder, and to understand how people with such disorders deal with work-related challenges	Hermeneutic phenomenology and reflexive methodology	Thirteen adults with experience of bipolar disorder who are receiving or have received treatment	Meaning and structure provided by work involving a variety of activities including the job Helpful roles and contexts outside illness provided by work—roles and contexts in which clients can use their skills, feel needed, and contribute Making work possible with support and help from others in one’s network Cost of working too much suggests work–rest balance; working too hard associated with clients’ initial episodes of mental health problems
Veseth, M. et al. [40]	Explore first person perspectives on identifying a bipolar disorder: how do individuals experience the process of discovering that they have a bipolar disorder? What does it mean for the person to find out that their symptoms and distress are in line with descriptions commonly seen as a severe mental illness?	Hermeneutic–phenomenological approach with individual in-depth interviews	Thirteen individuals with recovery experiences	Three phases of recovery: (a) “uncertainty and confusion” through (b) “grasping the novel and unusual experiential states” to (c) “giving meaning to the lived experiences of intense ups and downs”
Sælør, K. T. et al. [41]	How do persons with co-occurring mental health and substance use (MHSA) problems experience hope? What inspires hope, according to persons experiencing MHSA problems?	Cooperative action research approach with individual semi-structured interviews	Nine persons with MHSA problems	Daring to believe that something better is possible You need something to hold on to when you are looking for the light at the end of the tunnel You need some people you can trust and who has faith in you You have to decide whether you want to go on or not
Biong, S. [42]	Wha are the personal narratives of recovery of persons with substance abuse problems?	Phenomenological narratives—written narratives	Fourteen persons with MHSA	Recovery as a long process and involving changes in significant aspects of the persons’ lives for the better: Different prerequisites for the recovery processes Improvement as: “Improvement is the distance between who I felt I was and who I feel I am.” Building capacity for change taking a long time, requiring patience Requires continuous work with oneself Recovery is a natural part of life Recovery in terms of meaningful everyday life Focus on resources and future Involves re-establishing social life and social relations
Semb, R. et al. [43]	To explore how young adults with co-occurring MHSA problems experience a sense of belonging in their local environment, and facilitators and barriers related to belonging	Hermeneutic–phenomenological approach with in-depth interviews	Seven young adult users	Cannot find anything to relate to in the mainstream Balancing between mainstream and outsider life Trying to get a stronger foothold in the mainstream
Storch, J. et al. [44]	Explore and describe recovery as experienced by young adults who live with co-occurring MHSA	Qualitative, individual interviews	Seven young adult service users of municipal community MHSA services	The person is more than the diagnosis Users and professionals create different identities Focusing on possibilities and resources
Veseth, M. et al. [45]	Explore therapists’ views of the processes of recovery in bipolar disorders	A reflexive, collaborative approach with semi-structured individual interviews	Twelve professional providers	A “puzzling given” (as a fact that is incomprehensible) related to the complexity, unpredictability, and irregular patterning of bipolar disorders, pointing to recovery as complex Users as the protagonists of the healing process—personal qualities and strength, being resilient, and developing personal strategies to deal with problems The heroic fighter does not always win—dealing with disappointments and fights lost; respecting users’ hard work when unsuccessful
Brekke, E. et al. [46]	Explore and describe recovery as experienced by persons living with co-occurring MHSA	Phenomenological individual interviews	Eight persons with recovery experiences	Four dimensions of recovery: Feeling useful and accepted Coming to love oneself Mastering life Emerging as a person Insecure and inadequate housing and limited solutions to financial problems as major obstacles to recovery
Karlsson, B. et al. [47]	To explore and describeservice users’ experiences with peer support relationships, support and collaboration	Hermeneutic–phenomenological approach with focus group interviews	Twenty-six service users with MHSA problems	Relationships and collaboration with peer supporter workers as positive Challenges in peer support relationships and collaboration in terms of creating hope, equality, trust, and freedom to be helpful in other ways than those employed by professionals
Sælør, K. T. et al. [48]	How do relatives of people with mental illness describe their experiences of hope?	Phenomenological, descriptive approach with focus group interviews	Fifteen relatives of people with mental illness	Basic hope as a basic attitude, as a fundamental resource in life and a universal human condition of life, in line with love Everyday hope as hoping for a little more improvement and as qualitatively “small” hopes; linked to processing guilt, related to environmental factors, and experiencing hope in relation to one’s family members’ life situation
Sælør, K. et al. [49]	Stories of hope and recovery in MHSA	Written narratives	Two men with experience of MHSA	Stories providing images of the self and a way of sharing oneself Stories as ways to move forward to opportunities for change and hope Stories carrying contradictions Stories manifested through telling and clarifying oneself Stories of hopelessness as the beginning of hopefulness Stories as sharing
Pettersen, H. et al. [50]	To examine the role of social relationships in reaching and maintaining stable recovery after many years of substance use disorders	Individual interviews, narrative analysis	Eighteen adult service users with at least five years of stable recovery	Putting things straight with oneself and those around oneself Becoming responsible through boundary-setting practices Experiencing a strong sense of duty
Semb, R. et al. [51]	What do young adults with co-occurring MHSA find challenging in relation to belonging in their local communities?	In-depth individual interviews	Seven young adult users of municipal MHSA services	The need to accept one’s life and its surrounding structures: accepting one’s life story, and accepting the rules Being caught between conflicting social worlds—a choice between belonging to outsider life or the mainstream Moral fumbling in choices and actions—unprepared to be full participants in the mainstream and faltering moral and emotional connections to the mainstream along a continuum of condemnation versus self-blame
Brekke, E. et al. [52]	To explore and describe first-person experiences of relational recovery in persons with MHSA conditions	In-depth individual interviews	Eight adult service users with MHSA problems at various stages of recovery	Social relationships viewed as both supportive and hindering recovery: Choosing one’s child (parenting as the motivation for recovery) Living with loneliness and a painful past Sacrificing everything for one’s partner Regaining trust and support
Ogundipe, E. et al. [53]	How do persons with co-occurring MHSA problems in supported housing experience belonging? How do residential support staff experience promoting a sense of belonging for this group?	Collaborative and reflexive individual interviewing and focus group interview	Residents of a supported housing facility and the staff	The experience of belonging in relation to the contribution of the community and contextual factors in supported housing, such as: I do not go to sleep in my pajamas (supported housing being a house rather than a home and a lack of sense of belonging) Do I have a choice? (experiences of belonging connected to choice and having resources to make decisions on one’s behalf) Be kind to each other (the meaning of living with others)
Trangsrud, L. J. et al. [54]	To explore embodying experiences of nature related to recovery in everyday life for persons with eating disorders	Hermeneutic–phenomenological approach with individual interviews	Eight persons with experience of eating disorders	Experiences of nature as accentuating feelings of calmness and an engagement of the senses Nature experienced as a non-judgmental environment that also provides room for self-care Meeting nature through one’s body, particularly one’s feet, facilitating contact with the body and challenging the body–mind dichotomy

## Data Availability

All the included studies are in Table 1.
